# Flexible Multi-Layer Semi-Dry Electrode for Scalp EEG Measurements at Hairy Sites

**DOI:** 10.3390/mi10080518

**Published:** 2019-08-04

**Authors:** Haoqiang Hua, Wei Tang, Xiangmin Xu, David Dagan Feng, Lin Shu

**Affiliations:** 1 School of Electronic and Information Engineering, South China University of Technology, Guangzhou 510641, China; 2 School of Computer Science, The University of Sydney, Sydney 2006, Australia

**Keywords:** EEG, semi-dry electrode, flexible electrode, multi-layer

## Abstract

One of the major challenges of daily wearable electroencephalogram (EEG) monitoring is that there are rarely suitable EEG electrodes for hairy sites. Wet electrodes require conductive gels, which will dry over the acquisition time, making them unstable for long-term EEG monitoring. Additionally, the electrode–scalp impedances of most dry electrodes are not adequate for high quality EEG collection at hairy sites. In view of the above problems, a flexible multi-layer semi-dry electrode was proposed for EEG monitoring in this study. The semi-dry electrode contains a flexible electrode body layer, foam layer and reservoir layer. The probe structure of the electrode body layer enables the electrode to work effectively at hairy sites. During long-term EEG monitoring, electrolytes stored in the reservoir layer are continuously released through the foam layer to the electrode–scalp interface, ensuring a lower electrode–scalp contact impedance. The experimental results showed that the average electrode–scalp impedance of the semi-dry electrode at a hairy site was only 23.89 ± 7.44 KΩ at 10 Hz, and it was lower than 40 KΩ over a long-term use of 5 h. The electrode performed well in both static and dynamic EEG monitoring, where the temporal correlation with wet electrode signals at the hairy site could reach 94.25% and 90.65%, respectively, and specific evoked EEG signals could be collected. The flexible multi-layer semi-dry electrode can be well applied to scalp EEG monitoring at hairy sites, providing a promising solution for daily long-term monitoring of wearable EEGs.

## 1. Introduction

An electroencephalogram (EEG) is the sum of the postsynaptic potentials that occur synchronously with a large number of neurons, which is viewed as an electrophysiological indicator that records brain activity. It is the overall reflection of voltage fluctuations in the cerebral cortex or scalp surface caused by ionic currents in brain nerve cells [1]. Because EEG signals contain a large amount of physiological and psychological information, and EEG monitoring devices present a high temporal resolution, EEG has excellent prospects in psychiatric diagnosis [2,3] and brain–computer interfaces (BCIs) [4,5]. As one of the most anticipated developmental directions of human–computer interactions in the future, BCI systems can conduct human–computer interaction through the implicit information contained in EEGs [4]. There have already been breakthroughs in several applications, such as robotic arm control [6,7,8], drowsiness driving detection [9] and emotion recognition [10,11,12,13,14].

Nowadays, the electrodes used for EEG acquisition are mainly wet electrodes, which limit the application of EEG-based devices, especially in everyday situations. Wet electrodes are widely used in clinical and scientific research because of their low electrode–scalp contact impedance (5–20 KΩ) and relatively stable signal acquisition [15], and are regarded as the gold standard EEG measurement device. However, skin pretreatment and a conductive gel are required before using wet electrodes. Conductive gel will become dehydrated and dry with the passage of the EEG’s acquisition time, which causes an instability in electrode–scalp impedance, therefore wet electrodes are not suitable for long-term monitoring [16]. These shortcomings severely limit the application of wet electrodes in wearable EEG monitoring.

For the sake of solving the problems of traditional wet electrodes and developing electrodes suitable for daily EEG acquisition, various types of dry electrodes have been extensively studied [17,18]. Dry electrodes mainly include two types of invasive and non-invasive ones. Invasive dry electrodes are often made by Microelectromechanical Systems (MEMS) [19] and can pierce the stratum corneum with microneedles to obtain a lower electrode–scalp contact impedance. The microneedles are usually nanometers or micrometers in diameter, made of hard metal or crystalline silicon, and are often coated with conductive materials. However, it can irritate the scalp and is associated with a risk of infection due to breakage of microneedles [16]. Non-invasive electrodes are often based on flexible materials with probe structures designed to go through the hairs to fully contact the scalp. The probes are usually made of conductive materials or coated with conductive coatings, such as gold, Ag/AgCl or copper [20,21,22,23,24]. Several studies have also presented wearable electrodes based on conductive textiles due to their excellent wearable comfort and low cost, and the electrodes are much easier to integrate into smart clothes [25,26,27]. In addition to the contact electrodes, there is a class of non-contact electrodes that do not come into direct contact with the scalp, equivalent to capacitive coupling with the skin. However, EEG signals recorded by non-contact electrodes can be easily affected by movements, hence these electrodes are rarely used in daily detection [28].

Therefore, it is difficult to design a simple and stable electrode for monitoring EEGs especially at hairy sites. Several teams have begun work on semi-dry electrodes to address this problem. Peng et al. proposed a semi-dry electrode composed of polydimethylsiloxane (PDMS) and porous Ti materials [29]. Li et al. invented a porous ceramic column electrode [30]. The operating principle of the semi-dry electrode is to place a liquid storage tank inside the electrode, where the electrolyte is released during monitoring to form an ion channel between the scalp and electrode, which ensures the stability of the electrode–scalp contact interface, and maintains a low electrode–scalp contact impedance. But current semi-dry electrodes are usually made of rigid materials, which makes them uncomfortable and difficult to use for long periods of time.

In this paper, a flexible multi-layer semi-dry electrode containing a flexible electrode body layer, foam layer and reservoir layer was proposed to monitor EEG signals. Our semi-dry electrode can effectively solve the disadvantages of wet electrodes, such as the troublesome preparation process, need to apply conductive gel, and difficulty in long-term monitoring. To validate the performance of the semi-dry electrode, the electrode–scalp impedance and its long-term stability were tested, and the EEG signals collected by the electrodes under static and dynamic conditions were evaluated. The experimental results showed that the electrode had a low electrode–scalp contact impedance and performed well in the EEG monitoring process, providing a new solution for wearable EEG acquisition. Section 2 introduces the design and manufacturing method of the electrode and the paradigms of test experiments. The results and discussion are presented in Section 3. Section 4 draws the conclusion.

## 2. Materials and Methods 

### 2.1. Multi-Layer Structure Design

**Figure 1:**
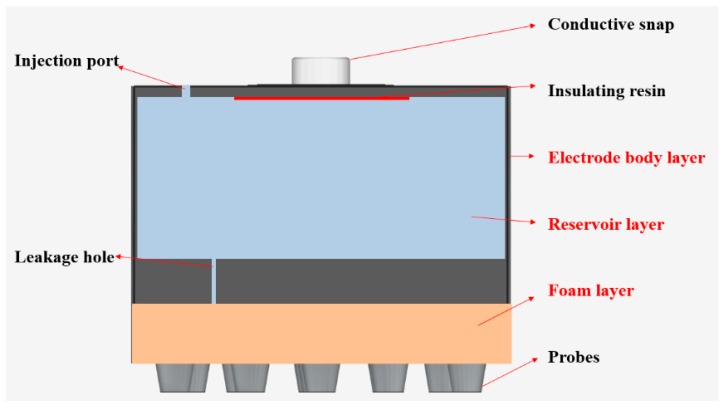
Structural diagram of the flexible multi-layer semi-dry electrode.

**Figure 2:**
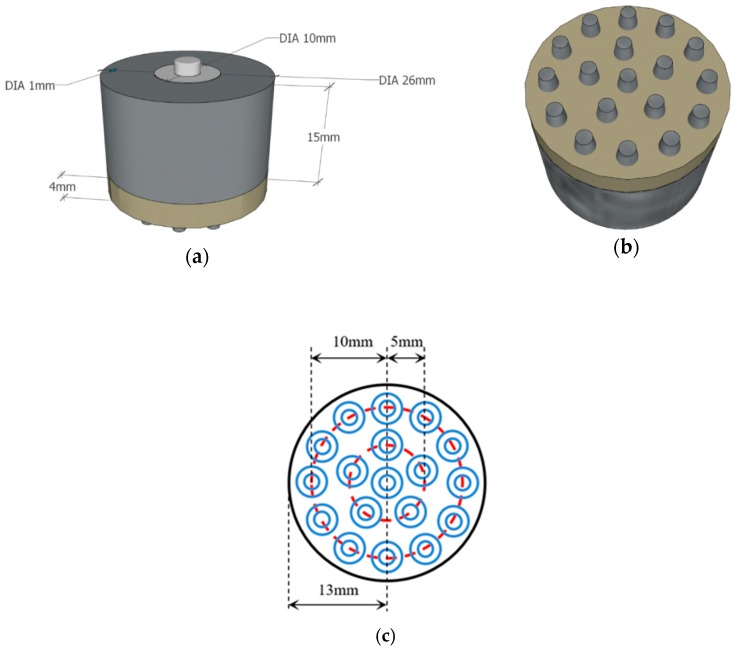
Design diagram of the flexible multi-layer semi-dry electrode: (**a**) front view of the electrode model; (**b**) bottom view of the electrode model; (**c**) schematic diagram of the probe distribution.

The flexible multi-layer semi-dry electrode consists of three layers of the electrode body layer, reservoir layer and foam layer, as shown in Figure 1. It is designed with 18 cone probes to ensure the electrode can reach the scalp through the hairs, where each probe is 6 mm high, 4 mm in diameter at the bottom and 2 mm in diameter at the top. The dimension of the electrode is 23 mm high and 26 mm in diameter and the foam layer height is 4 mm. The size of the electrode and the distribution position of the probe are shown in Figure 2. The electrode body layer contacts the scalp directly by the probes, forming the electrode–scalp contact interface. The electrode body layer is a hollow structure, and the internal cavity is the reservoir layer. Electrolyte solution can be added into the reservoir layer through the 1 mm diameter injection port at the top of the electrode, and the injection port will be plugged with an insulated PDMS plug when used. The electrolyte solution flows into the foam layer through the 5 leakage holes which are 0.25 mm in diameter at the bottom of the electrode. The foam layer stores the electrolyte so that it does not rapidly drain, ensuring stability of the electrode–scalp contact interface and low contact impedance. At the same time, since the foam layer is made of conductive foam, the high flexibility of foam allows it to contact the scalp to a certain extent, and also contributes to the acquisition of EEG. Three layers work together to ensure a lower electrode–scalp contact impedance, enabling a promising way of EEG measurement at hairy sites.

### 2.2. Materials and Fabrication

**Figure 3:**
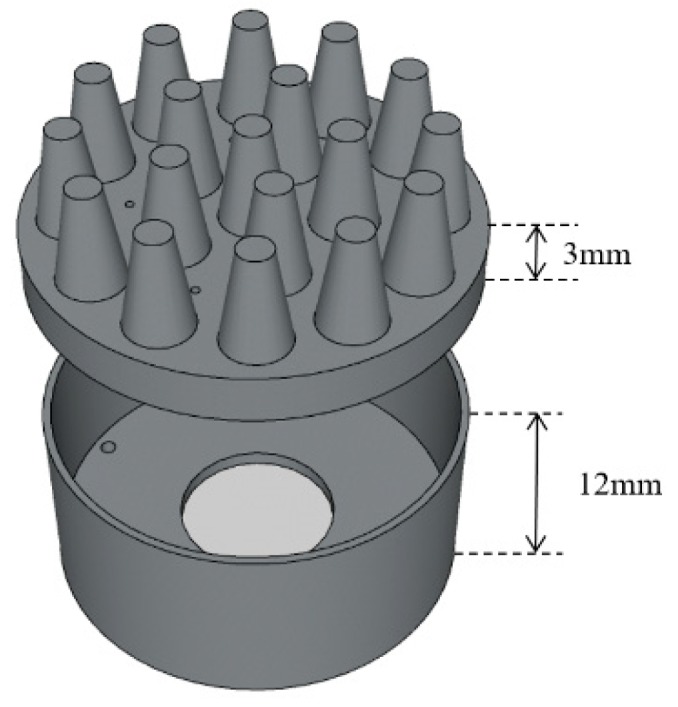
Schematic diagram of the electrode body layer.

**Figure 4:**
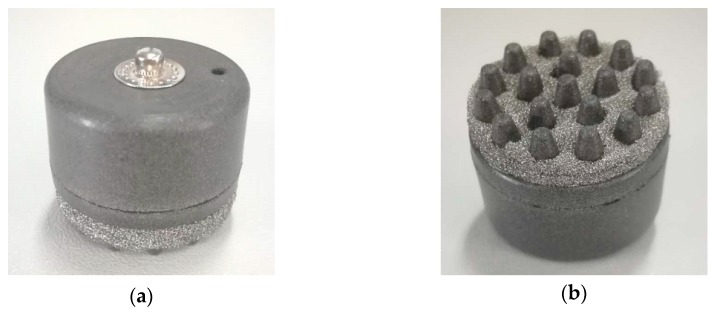
The flexible multi-layer semi-dry electrode: (**a**) top view of the electrode; (**b**) bottom view of the electrode.

In order to balance wearable comfort and EEG acquisition performance, the electrode body layer is made of flexible conductive composite materials with PDMS as the distributed matrix and silver nanoparticles as the conductive filler, in which the mass fraction of silver is 40 wt%. PDMS is a biocompatible, easily processed silicone elastic material. Silver nanoparticles have good electrical conductivity and stable electrical properties. The electrode layer is divided into two parts, as shown in Figure 3. The two parts were processed and formed in different molds, and were bonded together by conductive adhesives. The cavity between the two parts is the reservoir layer. The conductive snap is separated from the reservoir by insulating resin to avoid the electrochemical reaction of the electrolyte in contact with the conductive snap and unnecessary electrochemical noise during the EEG acquisition process. The conductive foam is coated with Cu and Ni nanoparticles, which makes the materials exhibit good electrical conductivity. Figure 4 shows the completed electrode.

### 2.3. Subject and Ethical Information

Ten subjects (ten males, age 23 ± 1 years, hair length of 5 ± 8 cm) took part in this study. All subjects were not on medication in the previous month, with no skin disease of the head, normal mental status, and no central nervous system abnormalities. All procedures have been approved by the Research Ethics Committee of the Body Data Science Engineering Center of Guangdong Province in China (BDS18-02). Informed consent was signed before the experiment began.

### 2.4. Electrode–Scalp Contact Impedance Measurement

**Figure 5:**
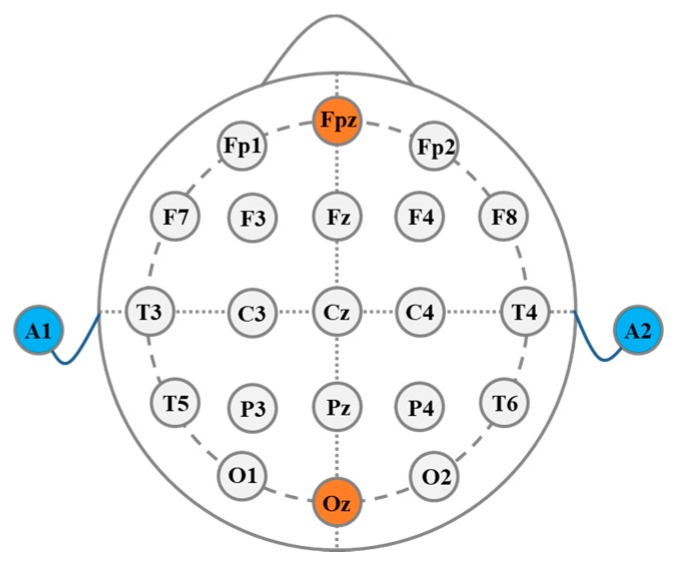
Schematic diagram of the electrode position.

The electrode–scalp contact impedance was measured using the electrochemical workstation (CHI660E, CH Instrument Inc., Austin, TX, USA) in a frequency range of 1–1000 Hz using a two-electrode system, and the distance between the two electrode centers was 4 cm. Two-electrode method was adopted to measure the electrode–scalp contact impedance using the electrochemical workstation with a 5 mV alternating current (AC) sinusoidal signal in a frequency range of 1–1000 Hz. The electrode position was in accordance with international 10/20 standard in this study, where A1 was the reference electrode position and A2 was the ground electrode position. The contact impedance of Fpz in the forehead and Oz in the back of the brain were measured five times for each subject, respectively, the positions of the experiment electrodes are shown in Figure 5. There was no scalp treatment during the experiment. Before the experiment, we used a syringe to add the electrolyte solution to the reservoir layer through the injection port. We chose 0.9% NaCl solution as electrolyte, whose composition was similar to human sweat [31], which had good electrical conductivity and could exchange the ion-electron current with the electrode. Since the pressure of the electrode on the scalp would affect the stability of the electrode–scalp contact interface and thus the electrode–scalp contact impedance, an adjustable elastic headband was used to fix the electrodes. In this study, the electrode pressure on the scalp was around 10 Pa. In order to test the long-term trend of the electrode–scalp contact impedance, the electrodes were fixed at the corresponding position and the impedance was measured hourly. During the long-term stability experiment, subjects were required to not touch the electrode and the elastic headband.

### 2.5. Long-Term Stability Experiment

A 5-h electrode–scalp contact impedance experiment was performed to evaluate the long-term stability of the electrode. The experimental setting was consistent with Section 2.4 and the electrodes were fixed at Oz. To obtain the trend of electrode–scalp impedance over time, the impedance at 10 Hz was measured per hour, because the 10 Hz alpha wave was the main rhythmic activity of the EEG. The subjects were required to not touch the electrode and the elastic headband during the experiment.

### 2.6. Static Electroencephalogram (EEG) Signal Evaluation

In this study, eyes close/open experiment and steady-state visual evoked potential (SSVEP) experiment were used to evaluate the EEG acquisition performance of the semi-dry electrode. The EEG signals were recorded by a neurofeedback and biofeedback system (NeXus-10, Mind Media, Herten, Germany) with a sampling rate of 256 Hz. The commercial disposable Ag/AgCl wet electrode used in the study was 10 mm in diameter.

#### 2.6.1. Eyes Close/Open Experiment

For each subject, we first measured their EEG at the Fpz, and then at the Oz. Disposable wet electrodes were placed next to the semi-dry electrodes for comparative analysis with the EEG collected from the semi-dry electrodes. During the experiment, the subjects were asked to close eyes for two minutes and maintain calmness, then open eyes and read an article for another two minutes. The correlation between the wet and semi-dry electrodes was calculated. According to the frequency characteristics of the scalp EEG, the signals were subjected to 0.1–40 Hz bandpass filtering. The EEG data was divided into three-second-long segments, then the temporal Pearson correlation between the EEGs collected by the semi-dry electrodes and wet electrodes was computed for every data segment, as follows:

(1) $$\rho_{X,Y}=\frac{\sum^{\text{​}}\left(X-\bar{X}\right)\left(Y-\bar{Y}\right)}{\sqrt{\sum^{\text{​}}{\left(X-\bar{X}\right)}^{2}\sum^{\text{​}}{\left(Y-\bar{Y}\right)}^{2}}}$$

X represents the amplitude of EEG voltage collected by the semi-dry electrode, and $\bar{X}$ is its average value in the data segment. Y represents the amplitude of EEG voltage collected by the wet electrode, and $\bar{Y}$ is its average value in the data segment.${\;\rho }_{X,Y}$ refers to the temporal Pearson correlation.

#### 2.6.2. SSVEP Experiment

SSVEP is a natural physiological feedback signal of the brain that corresponds to external visual stimuli. When a visual stimulus is presented at a fixed frequency, the human visual cortex responds to the stimulus frequency, which appears as an increase of EEG power at the stimulus frequency (integer multiples of the stimulus frequency). In this experiment, the semi-dry electrode was placed at the Oz, and visual stimulus was performed by a white LED which was 50 cm from the front of the subject at different frequencies (20 Hz, 14 Hz, 12 Hz, and no flashing lights).

### 2.7. Dynamic EEG Evaluation

**Figure 6:**
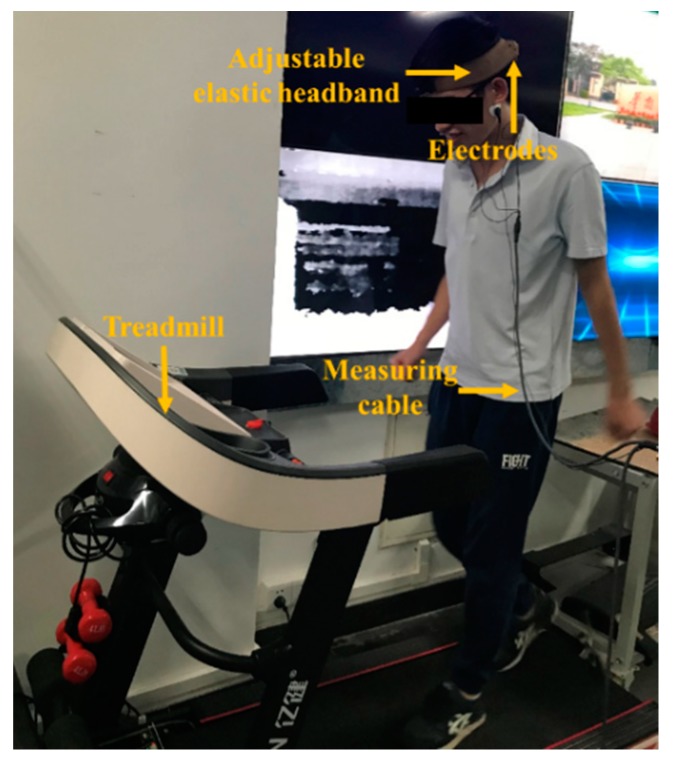
Dynamic electroencephalogram (EEG) measurement experiment setting.

In the case of motion, the acquisition of EEG signals will be greatly interfered because of the instability of the electrode–scalp contact interface [32,33]. To research the effect of motion artifacts on the semi-dry electrodes, the subjects were asked to jog at a speed of 10 km/h on a treadmill, with electrodes fixed near the Oz. During the experiment, EEG signals collected by the disposable wet electrode and the semi-dry electrode were simultaneously recorded, as shown in Figure 6.

## 3. Results and Discussion

### 3.1. Electrode–Scalp Contact Impedance of the Semi-Dry Electrode

**Figure 7:**
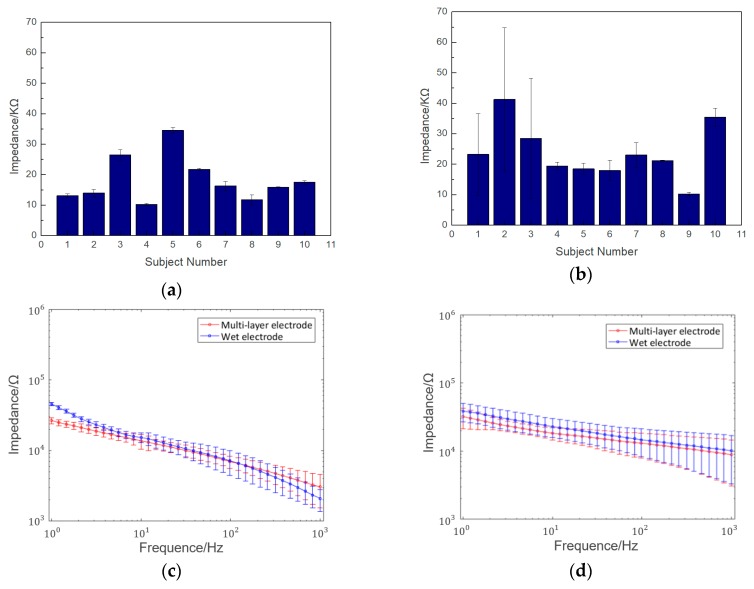
Results of semi-dry electrode–scalp contact impedance experiment: (**a**) impedance of 10 subjects at the forehead (Fpz) at 10 Hz; (**b**) impedance of 10 subjects at the back of the head (Oz) at 10 Hz; (**c**) frequency–impedance spectra comparison of the semi-dry and wet electrodes at the Fpz; (**d**) frequency–impedance spectra comparison of the semi-dry and wet electrodes at the Oz.

Figure 7a,b show the electrode–scalp contact impedance experimental results at 10 Hz for each subject at the Fpz and Oz. The average impedance at the Fpz and Oz points was 18.18 ± 7.51 KΩ and 23.89 ± 7.44 KΩ, respectively. The impedance at the Oz is higher than Fpz due to the hair shading and poor contact between the electrodes and scalp. Figure 7c,d show the frequency–impedance spectra of the semi-dry and wet electrodes at the Fpz and Oz, respectively. The results clearly showed the average electrode–scalp contact impedance of the semi-dry electrode at both the Fpz and Oz positions was a little lower than that of the wet electrode at frequencies below 50 Hz, which is the main frequency range of the EEG. Additionally, the electrode–scalp impedance of the electrode at the Oz was lower because the probe structure could pass through the hair, making the electrode–scalp contact area larger. While the disposable wet electrode is flat structure, and is thus difficult to directly attach to the scalp.

### 3.2. Long-Term Stability of the Semi-Dry Electrode

**Figure 8:**
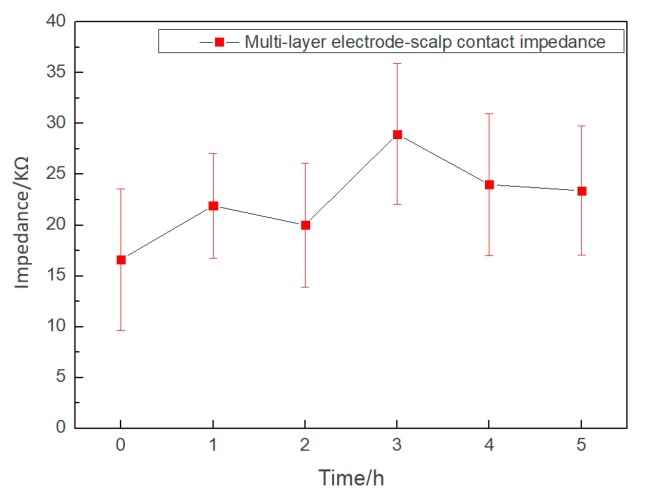
Changes of the semi-dry electrode–scalp contact impedance during the 5-h experiment.

Long-term experimental results of the electrode–scalp contact impedance at 10 Hz for 5 h are shown in Figure 8. The conductive gel of the wet electrode will gradually dry, and the electrode–scalp contact impedance at forehead will increase from 5 KΩ to 15 KΩ after 5 h of EEG monitoring, the impedance increased by about 200% [34]. During the experiment, the electrode–scalp contact impedance showed obvious fluctuations, this might be due to the complexity and interference of the electrode–scalp interface in the hair region. However, the impedance was always lower than 40 KΩ, which indicated that the electrode could effectively collect EEG during this period. At the end of the experiment, the foam layer remained moist, indicating that the electrolyte had not completely dried up.

### 3.3. Static EEG Signal Evaluation

#### 3.3.1. Results of Eyes Close/Open Experiment

**Figure 9:**
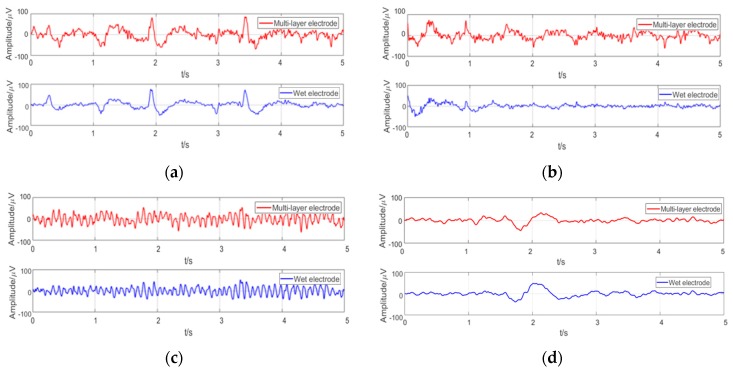
Typical EEG collected under different conditions in the eyes close/open experiment: (**a**) EEG at the Fpz with closed eyes; (**b**) EEG at the Fpz with opening eyes; (**c**) EEG at the Oz with closed eyes; (**d**) EEG at the Oz with opening eyes.

**Figure 10:**
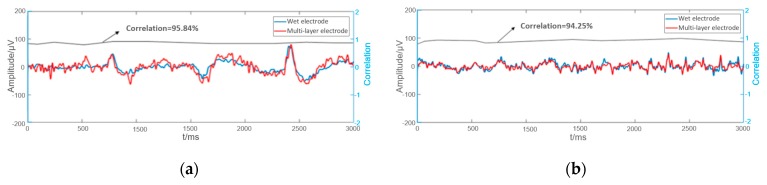
The correlation of the EEG signals collected by multi-layer semi-dry electrode and wet electrode: (**a**) EEG signals at the Fpz; (**b**) EEG signals at the Oz.

Some examples of EEGs acquired simultaneously by the multi-layer semi-dry and wet electrodes in the eyes close/open experiment are shown in Figure 9. The EEG signals collected simultaneously by the two kinds of electrodes at the same positions are similar. In order to compare the signal similarity collected by the wet electrode and the semi-dry electrode so as to better evaluate the EEG acquisition capability of the semi-dry electrode, a Pearson correlation analysis was performed on the signals. As shown in Figure 10, the average temporal correlation between the EEG collected by semi-dry electrode and wet electrode was 95.84% at the Fpz (subjects were asked to open their eyes and could blink during the process), and it can reach 94.25% at the Oz. This result indicates that the performance of the flexible multi-layer semi-dry electrode for collecting EEGs in this state is almost equivalent to that of the wet electrode.

#### 3.3.2. Results of SSVEP Experiment

**Figure 11:**
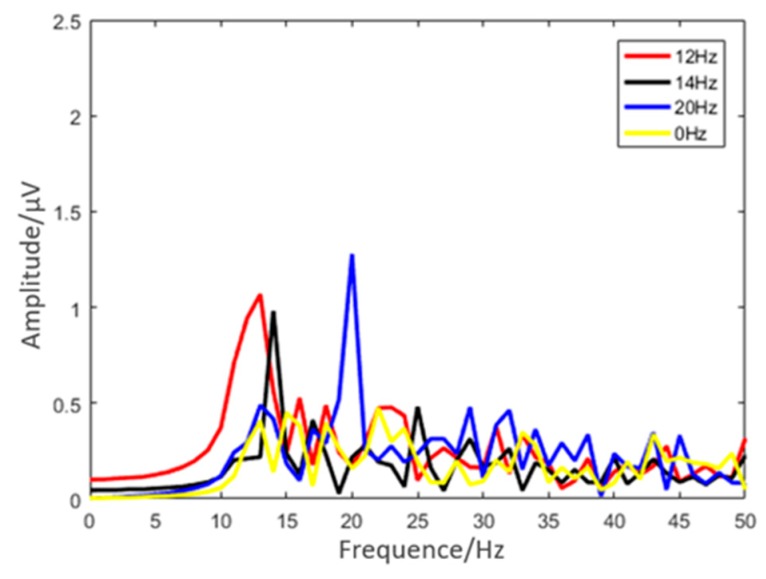
Results of the SSVEP experiment under different stimulus frequencies (12 Hz, 14 Hz, and 20 Hz).

The EEG spectra collected at the Oz in the SSVEP experiment of typical subjects are shown in Figure 11. There were clear spectral peaks corresponding to all three stimulus frequencies, and different SSVEP response peaks were obviously related to corresponding stimulus conditions. There are no strong peaks when the stimulus frequency is 0 Hz. The result shows that the flexible multi-layer semi-dry electrode can effectively measure the characteristics of SSVEP.

It can be seen from the experiments that, compared with commercial wet electrodes, the multi-layer semi-dry electrode can obtain reliable EEG signals in static test conditions, and the signal quality is nearly close to that of the wet electrode.

### 3.4. Dynamic EEG Signal Evaluation

**Figure 12:**
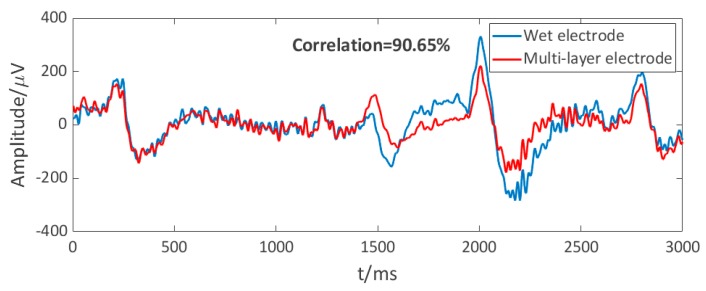
Dynamic EEG signals of the multi-layer electrode and wet electrode.

The electrode–scalp interface is unstable under active conditions, leading to motion artifacts. The amplitude of some motion artifacts overlaps with EEG, so it is difficult to clearly distinguish the artifact at present. Figure 12 shows the EEGs recorded by the semi-dry and disposable wet electrodes while jogging, which both involved the motion artifacts. Detecting EEGs in hairy areas has been a challenging target [35], since the wet electrode is the most widely used EEG electrode at present, the performance of the semi-dry electrode under motion was evaluated by calculating the correlation between the semi-dry electrode and the wet electrode signal, as shown in Figure 12. The correlation between the two EEG signals recorded by the semi-dry and wet electrodes in one motion test was 90.65%, which proves the EEG acquisition performance of the semi-dry electrode under motion is similar to that of the wet electrode. However, the disposable wet electrode is not ideal for dynamic EEG monitoring in hairy areas, because wet electrodes are difficult to fix in hairy areas and the electrode–scalp interface is very unstable in motion. Therefore, it does not mean that the performance of our semi-dry electrode is necessarily inferior to that of the wet electrode. In the future work, it is necessary to study an appropriate motion artifact recognition algorithm to better evaluate the acquisition performance of the semi-dry electrode in motion states.

### 3.5. Comparison with Other Electrodes

In order to better evaluate our semi-dry electrode, we made a detailed comparison with other types of electrodes, including a disposable wet electrode, other typical semi-dry electrodes and a dry electrode. As can be seen from the Table 1, the performance of our dry electrode was very similar to that of the disposable wet electrode. The electrode–scalp impedance of our semi-dry electrode was much lower than that of the dry electrode. Compared with other semi-dry electrodes, our semi-dry electrode used the lowest concentration of electrolyte but had a lower electrode–scalp impedance. The static EEG acquisition performance of our electrode in hairy site was closest to that of wet electrodes, and the dynamic EEG signal correlation with the wet electrode reached 90.65%, which also indicated that our electrode has great application potential in daily wearable EEG monitoring.

## 4. Conclusions

A flexible multi-layer semi-dry electrode which is suitable for dynamic long-term scalp EEG measurement has been developed and evaluated in this study. The probe structure makes it suitable for EEG monitoring in some hairy areas, such as the Oz. The multi-layer structure makes the electrode–scalp interface and the impedance be stable during long-term EEG monitoring processes. The semi-dry electrode is easy to operate and shows good acquisition performance in both static and dynamic EEG monitoring. As a kind of non-invasive electrode, semi-dry electrodes have a lower electrode–scalp impedance than dry electrodes, and also overcomes the disadvantages of traditional wet electrodes such as poor long-term monitoring performance, cumbersome operation, and scalp contamination. On the whole, the flexible multi-layer semi-dry electrode provides an appropriate solution for long-term EEG monitoring, which makes it have broad application prospects in wearable interactions and smart wearable health monitoring. In the future work, we will evaluate the performance of the electrode in more hairy areas over central and parietal sites and focus on developing electrodes suitable for whole-scalp EEG monitoring.

## Figures and Tables

**Table 1:** Comparison with other electrodes.

Electrode	Electrode Materials	Electrolyte	Electrode–Scalp Impedance at 10 Hz	Long-Term Impedance	Static EEG Signals Correlation with Wet Electrode	Dynamic EEG Signals Correlation with Wet Electrode
Disposable wet electrode	Ag/AgCl	None	19.03 ± 5.37 KΩ at Fpz (hairless), 24.66 ± 7.92 KΩ at Oz (hairy)	/	/	/
Multi-layer semi-dry electrode in this paper	PDMS, Ag nano-particles, conductive foam	0.9% NaCl solution	18.18 ± 7.51 KΩ at Fpz (hairless), 23.89 ± 7.44 KΩ at Oz (hairy)	Fluctuated between 11 KΩ and 40 KΩ in a 5 h test period (Oz)	95.84% at Fpz, 94.25% at Oz	90.65% at Oz
Passive ceramic based semi-dry electrode [29]	Al_2_O_3_ porous ceramic, Ag/AgCl	3.5% saline	42.1 ± 16.4 KΩ at O1 (hairy) ~ 51.4 ± 21.8 KΩ at O2 (hairy)	About 50 KΩ after 5 h and increased by approximately 20 KΩ in 8 h	93.80% ± 3.70%	Not mentioned
Quasi-dry electrode [36]	Polyurethane, Ag/AgCl	A NaCl solution of unknown concentration	Not mentioned	Not mentioned	The average of the four positions (Fp1, Fp2, O1 and O2) was 0.71	Not mentioned
Dry polymer electrode [37]	Polymer coated by Ag particles	None	About 80 KΩ in hairy site	Not mentioned	Not mentioned	Not mentioned
